# Antimicrobial Activity of Neutrophils Against Mycobacteria

**DOI:** 10.3389/fimmu.2021.782495

**Published:** 2021-12-23

**Authors:** Heather A. Parker, Lorna Forrester, Christopher D. Kaldor, Nina Dickerhof, Mark B. Hampton

**Affiliations:** Centre for Free Radical Research, Department of Pathology and Biomedical Science, University of Otago, Christchurch, New Zealand

**Keywords:** phagosomes, neutrophil extracellular traps, oxidative stress, tuberculosis, leprosy

## Abstract

The mycobacterium genus contains a broad range of species, including the human pathogens *M. tuberculosis* and *M. leprae*. These bacteria are best known for their residence inside host cells. Neutrophils are frequently observed at sites of mycobacterial infection, but their role in clearance is not well understood. In this review, we discuss how neutrophils attempt to control mycobacterial infections, either through the ingestion of bacteria into intracellular phagosomes, or the release of neutrophil extracellular traps (NETs). Despite their powerful antimicrobial activity, including the production of reactive oxidants such as hypochlorous acid, neutrophils appear ineffective in killing pathogenic mycobacteria. We explore mycobacterial resistance mechanisms, and how thwarting neutrophil action exacerbates disease pathology. A better understanding of how mycobacteria protect themselves from neutrophils will aid the development of novel strategies that facilitate bacterial clearance and limit host tissue damage.

## Introduction

Mycobacterium is a diverse genus comprising almost 200 species (1). The most well-known members are the human pathogens *Mycobacterium tuberculosis* and *Mycobacterium leprae*, which are the causative agents of tuberculosis and leprosy, respectively. Tuberculosis is a pulmonary disease that has plagued humans for thousands of years, and while global prevalence was reduced in the early 20th century due to the development of vaccines and antibiotics, the incidence has increased again such that it is estimated that a quarter of the world’s population is currently infected with *M. tuberculosis* with more than 4,000 deaths per day (2). The prevalence of leprosy is still of significant concern in endemic areas (3), and while curable the age-old stigma associated with leprosy still persists, creating fear and a reluctance to seek medical help. The mycobacterium genus also contains obligate and opportunistic pathogenic mycobacteria, which are grouped together as non-tuberculous mycobacteria (NTM). The incidence of NTM infection is increasing, such that in the USA the prevalence of pulmonary disease due to NTM is now greater than that of tuberculosis (4). The appearance of multi-drug resistant mycobacteria is of major concern, and new treatments are urgently required.

**Figure 1:**
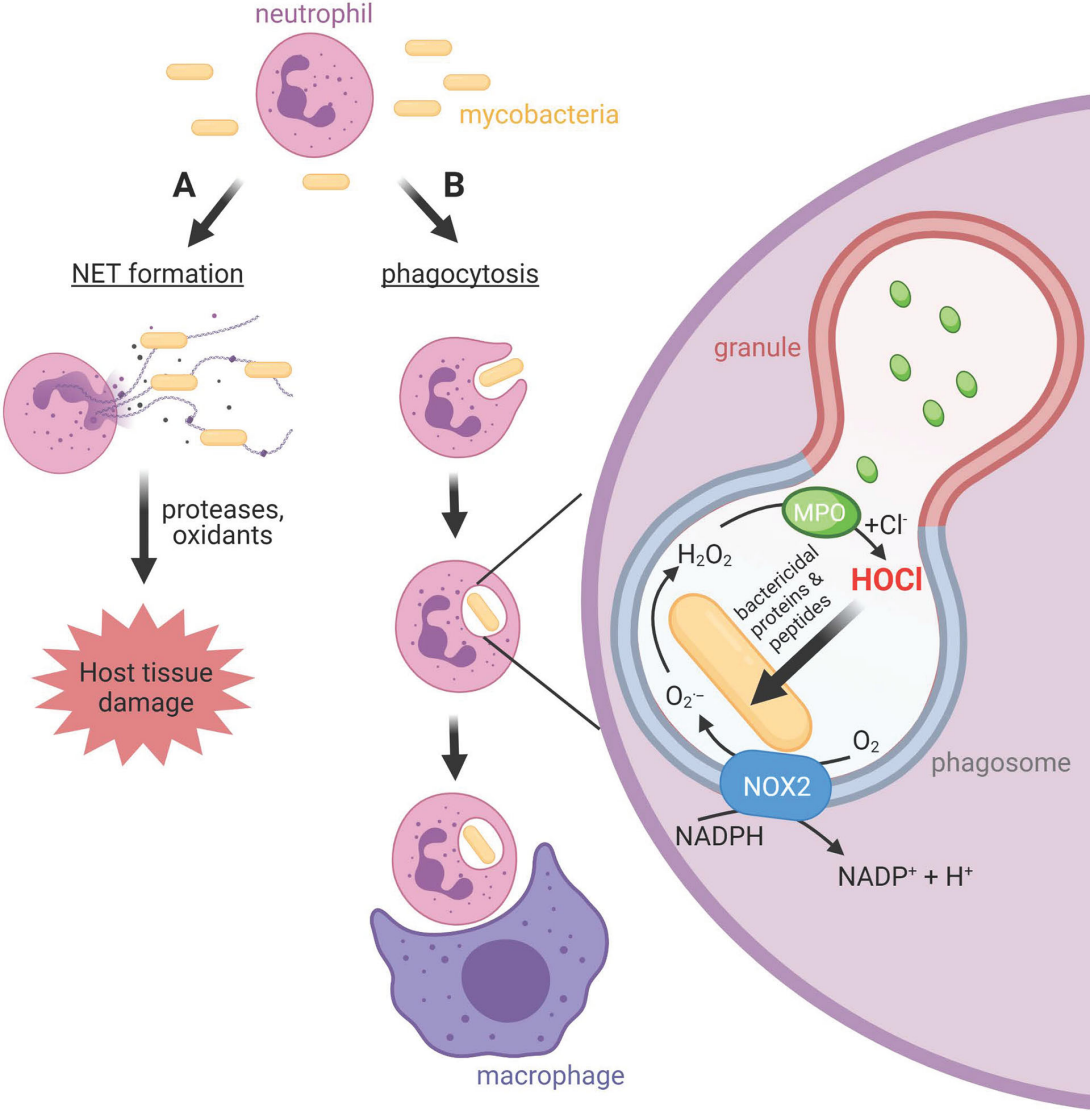
Neutrophil activities at sites of mycobacterial infections. **(A)** Neutrophils release neutrophil extracellular traps (NETs), chromatin structures decorated with neutrophil bactericidal peptides and proteins, in response to mycobacteria. Whether NETs contribute to bacterial clearance or predominantly promote host tissue damage is unclear. **(B)** Neutrophils phagocytose mycobacteria, albeit slower than other bacteria. The phagosomal membrane fuses with cytoplasmic granules to release antimicrobial peptides and proteins including myeloperoxidase (MPO) into the phagosome. The NOX2 assembles on the phagosomal membrane resulting in the production of superoxide and hydrogen peroxide, which MPO uses to produce the strong antimicrobial oxidant HOCl. Unlike other bacteria, and for reasons as yet unknown, mycobacteria do not succumb to HOCl produced in the phagosome. Neutrophils and their resident mycobacteria are ingested by macrophages. This may augment macrophage killing of mycobacteria *via* delivery of neutrophil antimicrobial agents, or provide transfer of live bacteria to a longer-lived host cell.

Mycobacteria can be remarkably successful intracellular pathogens that not only survive the initial assault of the innate immune system, but eventually take up residence within macrophages (5, 6). Neutrophils are also prominent in the lungs of patients with active pulmonary tuberculosis (7). They migrate to sites of infection in response to chemotactic signals, where they ingest pathogens into intracellular phagosomes (**Figure 1**). Neutrophil cytoplasmic granules fuse with the phagosomal membrane and empty antimicrobial peptides and proteins onto the pathogen, in a process termed degranulation. At the same time, an NADPH oxidase (NOX) complex assembles on the phagosomal membrane, transferring electrons from cytosolic NADPH to molecular oxygen in the phagosome. The initial product is superoxide, which dismutates to hydrogen peroxide and is converted to the potent bactericidal oxidant hypochlorous acid (HOCl) by another granule constituent, myeloperoxidase (MPO) (8). Pathogens that survive the initial oxidative burst may take up residence inside neutrophils. However, the neutrophil is a short-lived cell, providing a transport route into resident macrophages that are charged with clearing apoptotic neutrophils.

While the internalization of pathogens limits exposure of host tissue to the toxic compounds produced by neutrophils, extracellular release can occur (**Figure 1**). This includes the ejection of strands of chromatin coated with neutrophil proteins, which form a meshwork termed neutrophil extracellular traps (NETs) (9–11). NETs have been shown to trap bacteria and fungi and are thought to contribute towards containment of infection (9, 12) and antimicrobial activity (9, 13, 14). However, NETs can cause damage to host cells and tissue, and NETs are linked with various pathological conditions and diseases (15, 16). Unresolved inflammation will damage lung tissue and provide further opportunity for bacterial expansion.

In recent years it has become clear that neutrophils have more complex roles in immune regulation, with their ability to produce and modify cytokines, and release extracellular vesicles (17), enabling significant crosstalk with adaptive immune cells (18). This review focuses, however, on the early interactions between mycobacteria and neutrophils, and we ask the question of how pathogenic mycobacteria avoid destruction by neutrophils. Insight into their underlying survival mechanisms may provide therapeutic strategies that tilt this balance in favour of the neutrophil, and enable the early resolution of infection.

## Neutrophils in Mycobacterial Infection and Disease

### Neutrophils in Tuberculosis

Lung resident macrophages are the first immune cells to encounter inhaled *M. tuberculosis*, and they contribute towards bacterial clearance (19, 20). Neutrophils are subsequently recruited to the site of infection. The number of circulating neutrophils increases in patients with active tuberculosis (21–23), and a rise in neutrophil-derived transcriptional signatures has been observed in blood from patients with active tuberculosis (24). A study of close contacts of patients with active pulmonary tuberculosis showed an inverse correlation between peripheral blood neutrophil counts and risk of *M. tuberculosis* infection (21), and depletion of neutrophils from whole blood *in vitro* increased *M. tuberculosis* growth (25), suggesting that neutrophils can play an active role in limiting infection. Indeed, more neutrophils than macrophages were observed to have intracellular *M. tuberculosis* in sputum, bronchoalveolar lavage (BAL) fluid and granulomas from patients with active pulmonary tuberculosis (7). However, the fate of *M. tuberculosis* phagocytosed by neutrophils is not clear.

*M. tuberculosis* can survive and replicate within macrophages (26–28), where they are hidden from the immune system (29). Neutrophils have been proposed to play a similar “Trojan horse” role for *M. tuberculosis* (30, 31). Some bacteria and parasites, including *Yersinia* *pestis* (32), *Chlamydia pneumoniae* (33) and *Leishmania major* (34) can survive within neutrophils, and the length of *M. tuberculosis* bacilli observed in BAL fluid and sputum from patients with active tuberculosis was noted to be similar to lengths observed in logarithmic phase cultures (7), consistent with bacterial survival.

Neutrophils undergo apoptosis at sites of infection and can be cleared by macrophages. In these cases, any viable intracellular bacteria in neutrophils will be transferred to macrophages, where they can replicate and modulate macrophage responses and function. While it has been shown that neutrophils provide a source of antimicrobial agents that potentiate macrophage killing of *M. tuberculosis* by delivery of their granule contents (35), the survival of *M. tuberculosis* within macrophages has been shown to be enhanced after ingestion of neutrophils containing the bacterium (36). Further evidence that neutrophils play a permissive role in *M. tuberculosis* transmission was recently demonstrated in a report showing that dead neutrophils have the capacity to mediate short range, aerosol transmission of viable *M. tuberculosis* aggregates (37).

Disease severity in tuberculosis-sensitive mice has been linked to the survival of *M. tuberculosis* within neutrophils (31, 38). Neutrophil phagocytosis of *M. tuberculosis* was greater in genetically-susceptible mice than in those more resistant to tuberculosis, but the majority of the bacteria remained viable within the neutrophils (31). Furthermore, in a study of *M. bovis* BCG infection following inoculation of bacteria into the ears of C57BL/6 mice, neutrophils were observed to transport BCG to the auricular draining lymph nodes early in infection (30). BCG-laden neutrophils penetrated the paracortex, an area that is rich in T-cells (39). Notably, the bacilli load at the infection site did not decrease, indicating that in this model neutrophils do not clear the infection. Neutrophils are reported to be capable of functioning as antigen presenting cells (40), and others have suggested that while neutrophils may not directly control *M. tuberculosis* growth, they promote migration of dendritic cells to the lung draining mediastinal lymph node, facilitating priming of the adaptive immune response (41). However, neutrophils may protect the phagocytosed bacteria from recognition by the immune system thus delaying the adaptive immune response. In a study by Abadie et al. live BCG were recovered from the lymph nodes in numbers that remained stable over two weeks, lending support to this scenario (30).

The host response to pulmonary *M. tuberculosis* infection involves formation of nodule-like structures in the lung called granulomas, which comprise an assemblage of various immune cells, including neutrophils (42). Granulomas contain infection by preventing dissemination of bacteria; however, they may also provide a niche for *M. tuberculosis* survival (43–45). Neutrophils in zebrafish infected with *M. marinum* have been shown to work in conjunction with macrophages in developing granulomas to contain infection (46). However, in active disease the granulomas develop into large inflammatory lesions, and neutrophil accumulation may play an important part of this process. In a non-human primate model of tuberculosis, greater neutrophil accumulation was observed in the larger granulomas associated with active tuberculosis compared with the smaller granulomas of a latent infection (47). In a mouse model, interleukin (IL)-17 neutralization decreased neutrophil accumulation in lung granulomas but there was no difference in bacterial burden (47), while neutrophil depletion in C57BL/6J mice during the chronic phase of infection improved control of *M. tuberculosis* (48).

Several studies suggest that neutrophil accumulation is associated with a dysregulated immune response and a poor prognosis in tuberculosis. Even after bacterial clearance, neutrophil accumulation is linked with post-TB lung disease (49). Levels of calprotectin, the most abundant neutrophil cytoplasmic protein, and neutrophil chemokines are increased in patients with active tuberculosis compared to healthy controls and those with latent infection, and this is correlated with lung damage (47). Increased neutrophil accumulation was shown to be mediated, at least in part, *via* calprotectin-dependent upregulation of the neutrophil integrin CD11b (48). Calprotectin has antimicrobial activity, however improved control of infection was observed when a subunit of calprotectin was knocked out (48). Mice infected by aerosol with a low dose of *M. tuberculosis* were grouped into three different classes: resistant, susceptible and super-susceptible mice; reflecting the variation observed in humans (50). Lungs of super-susceptible mice showed the highest numbers of infiltrating neutrophils, had the largest granulomas with areas of necrosis, and the highest bacterial burden. Another mouse study found that neutrophil depletion significantly increased survival following *M. tuberculosis* infection (51). Using several different models, Mishra et al. concluded that neutrophil accumulation in the lungs correlated with increased bacterial burden and animal weight loss, and that inhibition of neutrophil recruitment decreased bacterial numbers (52).

In summary, current evidence indicates that neutrophils play an important role during in bacterial clearance the acute stages of human TB. This is supported by several animal studies (53, 54). However, if the infection continues, accumulating evidence indicates that neutrophils contribute towards disease pathology and a negative prognosis. Investigation of the suitability of neutrophils as a marker for poor outcome in TB is ongoing (55).

### Neutrophils in Leprosy

*M. leprae* infects macrophages (56) and Schwann cells (57), and it is these cells that are traditionally thought to be important players in infection (58, 59). Peripheral blood neutrophils from lepromatous leprosy patients have, however, also been shown to harbor *M. leprae* (60). The course of lepromatous leprosy can involve periods of acute inflammation called reactions, the most common of which is erythema nodosum leprosum (ENL). Neutrophil infiltration is a feature of the skin lesions associated with this reaction, although neutrophils are not always present (61–63). ENL can occur as a single acute episode, several discrete acute episodes or as a chronic condition, and can occur before, during or after multi-drug therapy (64, 65). Importantly, ENL is a major contributor towards nerve damage in leprosy patients and contributes significantly towards mortality (66). Despite the fact that neutrophils are the hallmark of ENL in histological samples (67), few studies have examined their role, but the evidence indicates neutrophils may make a significant contribution to the pathogenesis of ENL [reviewed in (63, 68, 69)].

Two early studies examined neutrophil activation in ENL by measuring reduction of nitroblue tetrazolium (NBT) (70, 71), which is reduced by superoxide to dark formazan precipitates (72). In the first study, spontaneous reduction of NBT was significantly increased in blood from patients with reactional lepromatous leprosy (RLL), of which ENL is a major subset, compared with blood from healthy controls and patients with other forms of leprosy (70). RLL patient sera did not increase neutrophil activation in blood from healthy controls, suggesting that the activating stimulus was not present in the circulation (70). In contrast, the second study that used isolated neutrophils reported no increase in spontaneous NBT reduction in neutrophils from ENL patients, and ENL patient sera induced a large increase in NBT reduction in neutrophils from healthy controls and ENL patients (71). The difference between studies may have been due to the use of heparin at higher concentrations, which can form particles with NBT that activate neutrophils (73), and/or the presence of an inhibitory factor that is absent from ENL sera.

Activated neutrophils express the Fcγ-receptor I (Fcγ-R1) (also known as CD64), a high affinity receptor for IgG (74). Fcγ-R1 expression was observed on neutrophils within ENL skin lesions and expression was significantly higher in the peripheral blood of ENL patients compared to lepromatous leprosy patients without ENL (62). The presence of Fcγ-R1 expressing neutrophils in peripheral blood increased with disease severity, and treatment of ENL with thalidomide, which improved symptoms, decreased Fcγ-R1 expression and the level of neutrophils in skin lesions (62). In addition, the neutrophil granule protein pentraxin-3 (PTX-3) was found to be increased in the blood of multibacillary leprosy patients, in particular, levels were higher in those that went on to develop ENL (75). PTX-3 levels correlated with Fcγ-R1 expression in the circulation and PTX-3 was also increased in ENL skin lesions and correlated with neutrophils (myeloperoxidase) (75). More studies on the relationship of neutrophils to more severe forms of leprosy are needed.

### Neutrophils in Other Mycobacterial Infections

*M. avium* complex (MAC) are the most common cause of NTM-induced pulmonary disease, predominately, but not exclusively, in those with pre-existing lung conditions (76). They are also a leading cause of disseminated NTM infection (76). Mouse studies examining the role of neutrophils in MAC infection show differences in their role dependent on the form of infection, pulmonary or systemic. C57BL/6 mice with the beige mutation, whose neutrophils are defective in chemotaxis and killing (77), have increased susceptibility to MAC infection (78, 79). In a study of disseminated infection, neutrophil transfusion from WT mice improved the resistance of beige mice to intravenous *M. avium* infection while neutrophil depletion in WT mice increased their susceptibility (78). This implies a protective role for neutrophils in systemic MAC infection in this mouse model. However, in lung infection of C57BL/6 mice, Saunders et al. found that a 95% decrease in neutrophils in the lungs had no effect on bacterial numbers, indicating neutrophils are dispensable in controlling MAC lung infection in these mice (79). Further support that neutrophils are ineffective in controlling MAC infection was provided by a study of mice over-expressing the transcription factor RAR-related orphan receptor gamma t (RORγt), which regulates Th17 responses and increased pulmonary neutrophil infiltration, yet bacterial burden was similar to that in WT mice (80).

In humans, a study of MAC-infected patients with no pre-existing lung disease found that the level of neutrophils in patient BAL fluid was significantly higher than that of control patients (81), with higher neutrophil counts subsequently correlated with worsening disease (82). Similarly, in a more recent retrospective study of pulmonary MAC infection, BAL fluid from patients whose disease progressed had higher numbers of neutrophils than those who had stable infection (83). Together these studies provide evidence that neutrophils are ineffective in preventing MAC-induced pulmonary disease.

Slow growing *M. kansasii* are considered to be the most pathogenic of the NTM, as when isolated they are almost always associated with disease (84, 85). *M. kansasii* most frequently cause pulmonary disease that is clinically similar to tuberculosis (86), but infections at other sites are also reported (87). In humans, abundant neutrophils have been observed at sites of *M. kansasii* infection (87–89). In CD-1 mice, peritoneal inoculation with *M. kansasii* or *M. avium* was found to lead to chronic neutrophil infiltration, with bacterial numbers gradually decreased during this time (90). Neutrophil ingestion of *M. kansasii* and *M. avium* was not examined, however neutrophil uptake of non-pathogenic *M. aurum* only occurred during the first two days (90). Macrophages were present at the infection site and ingested dying neutrophils (90). Lactoferrin, present in neutrophils but not macrophages, was detected within peritoneal macrophages suggesting transfer from neutrophils to macrophages either by neutrophil degranulation and subsequent uptake of granule components by resident macrophages, or through uptake of the intact neutrophil. Macrophage antibacterial activity *in vitro* was enhanced when the macrophages were pre-incubated with neutrophils, leading the authors to conclude that neutrophils do not directly control NTM infection but participate indirectly *via* transfer of macromolecules that enhance macrophage killing of these bacteria (90).

*M. abscessus* are rapidly growing NTM that cause pulmonary disease in both healthy individuals and those with underlying lung disease, disseminated infections, and skin and soft tissue infections (91–93). Of note, *M. abscessus* are particularly recalcitrant to antibiotic therapy (94, 95). Extensive numbers of neutrophils are reported within patient granulomas or at the site of infection (96–98). In addition, human lung tissue infected with *M. abscessus ex vivo* showed bacteria within neutrophils at the site of infection (99). *M. abscessus*, like several other NTM (91), exist in one of two colony morphological forms. A smooth, non-cording form and a rough cording form, that differ in their concentration of cell wall glycopeptidolipid (100). The rough form is associated with more severe pulmonary disease (101). Greater neutrophil numbers were measured in BAL fluid from C57BL/6 mice infected with the rough form (102). In a zebrafish model of *M. abscessus* infection, ingestion of the rough form by macrophages was associated with increased macrophage apoptosis, release of viable bacteria and intense cording growth, which neither macrophages nor neutrophils could engulf because of the size of the cords (103). However, in a subsequent study both rough and smooth forms induced a large influx of neutrophils early at the infection site that was dependent on IL-8 and macrophage-secreted TNF (104). Both forms were engulfed equally by neutrophils, and neutrophil depletion resulted in uncontrolled bacterial growth and zebrafish larvae death with either (104). Neutrophils were found to be essential for the development and maintenance of protective granuloma in the infected zebrafish (104).

*M. smegmatis* is a rapidly growing NTM that is ubiquitous in the environment and is generally considered to be non-pathogenic. Due to its fast replication rate (relative to other mycobacteria), amenability to genetic manipulation, and the fact that it can be grown under normal Biosafety Level 2 laboratory conditions, *M. smegmatis* is often used as a model to study *M. tuberculosis* infection and virulence factors. Occasionally *M. smegmatis* causes skin and soft tissue infections, and very rarely disseminated infection (105–108). Hospital-acquired infections also occur, resulting from a variety of procedures including catheterization, cardiac and plastic surgery (109). Although infrequent, these infections can be difficult to treat requiring surgical debridement and long term antibiotic therapy (105). Neutrophils are recruited to the site of infection in humans (106, 110), and in mice infected with *M. smegmatis* intratracheally (111). *M. smegmatis* has been shown to induce neutrophil exocytosis of gelatinase granules releasing active matrix metalloproteinase-9 that degrades the extracellular matrix (112). Release of these granules may contribute towards the tissue degradation observed in soft tissue infections caused by this bacteria. Neutrophil exocytosis of azurophilic granules has also been reported in response to *M. smegmatis* and constituents of these granules can also cause host tissue damage (113).

Much work remains to be done to gain a better understanding of the role of neutrophils in mycobacterial infection and disease. The plethora of studies implicating neutrophils in the pathogenesis of mycobacterial infections strongly suggests that the capacity of neutrophils to control infection by intra- and extra-cellular killing mechanisms is either insufficient, defective or thwarted by mycobacteria. In the next sections of this review we discuss the current evidence for neutrophil phagocytosis and killing of mycobacteria (both phagosomal and NET-mediated) and evidence for resistance of mycobacteria to neutrophil oxidants.

## Neutrophil Phagocytosis of Mycobacteria

The major antimicrobial strategy employed by neutrophils involves the ingestion of pathogens into phagosomes, followed by the degranulation of antimicrobial peptides and proteins and the production of toxic reactive oxygen species inside the phagosome (8, 114). Neutrophils are known to phagocytose both pathogenic and non-pathogenic mycobacteria (7, 30, 36, 112, 115–121), with ingestion of mycobacteria increasing in the presence of serum (112, 115–117). The mechanism of phagocytosis differs depending on whether neutrophils bind opsonized or non-opsonized mycobacteria. Neutrophil complement receptor 3 (CR3) binds *M. leprae* phenolic glycolipid-I resulting in bacterial phagocytosis and activation of the Syk tyrosine kinase, which leads to activation of the transcription factor NFATc and Il-10 production (122). Phagocytosis of non-opsonized *M. kansasii* has also been shown to occur *via* CR3 in a cholesterol-dependent and glycosylphosphatidylinositol (GPI) anchored protein-dependent manner, while cholesterol was not required with opsonized bacteria (123). Phagocytosis of non-opsonized *M. smegmatis* was also dependent on CR3 and cholesterol (113). GPI-anchored proteins and cholesterol accumulate in lipid rafts (124) suggesting that the localization of CR3 to lipid rafts is required for neutrophil internalization of non-opsonized mycobacteria. The glycosphingolipid lactosylceramide (LacCer), enriched in lipid rafts (125), is also required for non-opsonic internalization of mycobacteria (126). Binding to LacCer is mediated by lipoarabinomannan (LAM) on the mycobacterial surface. Interestingly, the mannose cap on LAM (ManLAM) of pathogenic mycobacteria, but not the phosphoinositol cap (PILAM) of non-pathogenic mycobacteria, appears to prevent fusion of azurophil granules with the phagosome (126), which will have a significant impact on the antimicrobial activity of neutrophils.

While most studies report that neutrophils phagocytose mycobacteria, a few have reported impaired phagocytosis. In zebrafish infected with *M. marium*, neutrophils were absent from the initial infection site but were recruited to the developing granuloma by dying macrophages (46). Neutrophils then phagocytosed *M. marium* indirectly by taking them up from macrophages. The investigators observed a small increase in direct neutrophil phagocytosis of *M. marium* when inoculum numbers were increased (46). Neutrophil uptake in BALB/c mice was shown to be negligible after intravenous injection of a relatively low dose of *M. tuberculosis* (53). However, Abadie et al. observed abundant neutrophil phagocytosis of BCG in C57BL/6 mice after inoculation with a similar low dose of bacteria as present in the BCG vaccine (30).

Recently we measured the rate of phagocytosis of *M. smegmatis* and found that it was five times slower than the phagocytosis of *Staphylococcus aureus* and 3.5 times slower than that for *Escherichia coli* (127, 128). Phagocytosis of *M. abscessus* was also found to be slower than that of *S. aureus* when examined by counting the number of neutrophils containing fluorescently labelled bacteria (129). In another study, approximately half of a population of *M. fortuitum* was phagocytosed within 30 min (130), only slightly faster than the 43 min we measured for *M. smegmatis*, and still considerably slower than the 9 min for *S. aureus* and 12 min for *E. coli* (127, 131). As far as we are aware no *in vitro* studies have directly compared phagocytosis of different mycobacteria by the same neutrophils; however, neutrophils were found to ingest *M. abscessus* more frequently than either *M. tuberculosis* or *M. avium* in an *ex vivo* infection of human lung tissue (99).

Evidence from tuberculosis patients indicates that patient neutrophils are primed for phagocytosis. Surface expression of the Ig receptor FcγR1 (CD64) was found to be increased on peripheral blood neutrophils from patients with tuberculosis pleuritis compared to healthy controls, and neutrophils obtained from pleural fluid showed further enhanced FcγR1 expression and increased expression of the pattern recognition receptor TLR2 (132). An increase in expression of FcγR1, TLR2 and TLR4 was also observed in neutrophils from the peripheral blood of patients with active pulmonary tuberculosis prior to treatment (133). Despite the evidence for increased receptor expression, studies examining the phagocytic activity of neutrophils from patients with active pulmonary tuberculosis generally show their capacity for phagocytosis is decreased. Hilda et al. measured significantly reduced phagocytic activity in blood neutrophils from patients prior to treatment (133), and Shalekoff et al. also found peripheral blood neutrophil function impaired in patients with active pulmonary tuberculosis (134). Patient neutrophils showed a reduced capacity for phagocytosis compared to healthy controls and this was observed both soon after the start of treatment and when treatment had been undertaken for almost 30 weeks (134). Similarly, another study found peripheral blood neutrophils from tuberculosis patients had reduced phagocytic activity compared to healthy controls (135). In these studies, phagocytic capacity was measured in patient neutrophils by assessing phagocytosis of *E. coli* or yeast to rule out *M. tuberculosis* factors that may interfere with neutrophil uptake.

Mycobacteria are able to directly inhibit neutrophil phagocytosis. *M. abscessus* rough morphotypes prevent phagocytosis by formation of serpentine cords that are too large for neutrophils to ingest, with these large aggregates linked to pathogenesis (103). *M. leprae* cell wall lipids inhibit macrophage phagocytic activity (136), but to our knowledge there are no reports on whether these lipids affect neutrophil phagocytosis. Exposure of *M. tuberculosis* to human alveolar lining fluid results in changes to the bacterial cell wall and release of cell wall fragments (137). Macrophage phagocytosis of *M. tuberculosis* decreased when the bacteria were pre-exposed to alveolar lining fluid, however, neutrophil phagocytosis was found to increase (138, 139). This increase in phagocytosis was due to bacterial alveolar lining fluid exposure rather than *M. tuberculosis* cell wall fragments released by treatment with alveolar lining fluid (139).

## Phagosomal Killing of Mycobacteria

The ability of neutrophils to kill *M. tuberculosis* is controversial with some studies observing killing (116, 140–142) while others do not (115, 143–145). Neutrophils have been reported to kill 50-70% of *M. tuberculosis* within 90-120 minutes (116, 140); however, Corleis et al. found neutrophils did not kill *M. tuberculosis* even after six hours co-incubation (115) and others found no killing after incubation with neutrophils for 24 hours (144, 145). While Hartman et al. reported near complete killing of *M. avium* by neutrophils in two hours (120), only around 40% of populations of *M. abscessus* and *M. fortuitum* were killed after two hours incubation with neutrophils (146). We have previously reported half-lives for *E. coli* and *S. aureus* inside the neutrophil phagosome of 2 min and 6 min, respectively (128), and recently measured a half-life for *M. smegmatis* of 30 min inside the neutrophil phagosome, which indicates that even though killing occurs, it is slow (127).

In terms of bactericidal mechanisms, the neutrophil oxidative burst is activated upon uptake of various mycobacteria, including *M. tuberculosis*, *M. canettii*, *M. abscessus*, *M. kansaii*, *M. phlei*, *M. fortuitum* and *M. smegmatis* (115, 117, 130, 144, 146–148). Contrary to this, *M. gordonae* did not stimulate neutrophil oxidant production (144) and *M. bovis* induced a weak oxidative burst in comparison to *Listeria monocytogenes* (118). Interestingly, *M. tuberculosis* were found to induce a stronger oxidative burst than *M. smegmatis*, yet *M. smegmatis* was killed while *M. tuberculosis* was not (115). Rough morphotypes of *M. abscessus* induced a stronger oxidative burst in comparison to smooth morphotypes, yet neutrophil killing of both was unaffected by inhibition of oxidant production (146).

The purified MPO/H_2_O_2_/Cl^-^ system is capable of killing *M. tuberculosis* and *M. leprae* (149, 150); however, it has been reported that the complete system did not augment killing of *M. tuberculosis* over that observed by H_2_O_2_ alone (141). Reagent HOCl killed *M. smegmatis*, but approximately seven times more HOCl was required to kill *M. smegmatis* than *S. aureus* suggesting mycobacteria may be innately more resistant to HOCl (127). Further studies are required to determine if other mycobacteria are similarly resistant to HOCl. We recently sought to examine whether HOCl plays a role in killing of *M. smegmatis* in the neutrophil phagosome. The amount of reagent HOCl required to kill a bacterium cannot be directly translated to the phagosome as neutrophil oxidants are produced in a flux in the phagosome, and a flux of HOCl may be less harmful to the bacteria than a single high dose. Additionally, the neutrophil phagosome contains many proteins and amines that can react with phagosomal HOCl before it reaches the bacterium (151, 152). Using a fluorescent probe we observed HOCl production in the phagosome and that MPO inhibition abrogated HOCl production (127). By modelling the data obtained in our study we estimated that it would take 30-40 minutes at full MPO capacity for sufficient HOCl to be produced to kill a single ingested *M. smegmatis*. Sustained MPO activity for that length of time is unlikely and therefore we concluded that insufficient HOCl is produced in the neutrophil phagosome to directly kill this bacterium. In support of this conclusion, inhibition of MPO had no effect on neutrophil killing of *M. smegmatis* (127).

Inhibition of the NADPH oxidase had no effect on the ability of human neutrophils to kill *M. tuberculosis* and *M. abscessus* (142, 146). Defective killing by neutrophils from patients with chronic granulomatous disease (CGD), who have mutations that lead to a non-functional NADPH oxidase (153), is often used as evidence for the requirement for oxidants in bacterial killing. Jones et al. showed that CGD neutrophils killed *M. tuberculosis* as effectively as neutrophils from healthy donors (140). However, in countries where tuberculosis is endemic, the incidence of tuberculosis is greater in CGD patients than the rest of the population (154–156), though it is important to consider that NOX2 has other roles during inflammation (157). X-linked CGD mice showed increased bacterial growth and an increase in granuloma size in their lungs compared to C57BL/6 mice (158). A high incidence of complications due to BCG vaccination has also been reported in CGD patients (155, 156, 159–161). Neutrophil killing of *M. marinum* was found to depend on an active NADPH oxidase in a zebrafish model of early tuberculosis disease using morphant larvae with neutrophils deficient in two subunits of the NADPH oxidase (*gp91^phox^* and *pg22^phox^*) (46). In a zebrafish model of cystic fibrosis, transmembrane conductance regulator morphants showed reduced neutrophil oxidant production and reduced intracellular control of *M. abscessus* that was linked to a reduction in NADPH oxidase activity (147).

Neutrophil killing of mycobacteria can occur *via* non-oxidative processes. Defensins, components of azurophil granules, have been shown to have anti-mycobacterial activity (21, 162, 163). Defensin-depleted granules have also been shown to kill *M. tuberculosis*, *M. bovis* BCG and *M. smegmatis* although *M. tuberculosis* were more resistant to killing (164). The azurophil granule proteins elastase, azurocidin, and lysozyme, and the specific granule protein lactoferrin were shown to kill *M. smegmatis* (164). In addition, conditioned media from neutrophils incubated with *M. abscessus* for 90 minutes showed bactericidal activity towards this bacterium (146). This was most likely due to degranulation of cytotoxic agents. *In vitro*, *M. tuberculosis* induced neutrophils to release MPO and elastase (165) and neutrophils were found to release elastase and cathepsin G, another major azurophil granule protein, into the bronchoalveolar space in mice infected with *M. bovis* BCG (166). Released neutrophil granule proteins have been shown to be taken up by infected macrophages and to increase macrophage killing of *M. tuberculosis* and *M. bovis* BCG (35, 164). Efferocytosis also potentiates macrophage killing of ingested *M. tuberculosis* through delivery of granule proteins to early endosomes and fusion of these with the phagosome (35). Augmented macrophage killing due to uptake of neutrophil granule proteins has been demonstrated in other bacteria (167, 168). When neutrophils struggle to control a mycobacterial infection through intracellular killing, degranulation and efferocytosis could be an important mechanism by which neutrophils contribute towards host defense.

In the early stages of *M. tuberculosis* infection, and when they escape from phagocytic cells, *M. tuberculosis* are exposed to a variety of agents in alveolar lining fluid (ALF) that can alter the bacterial cell wall (137). Exposure of neutrophils to *M. tuberculosis* pre-treated with human ALF increased phagocytosis and neutrophil killing of *M. tuberculosis* while dampening the oxidative burst response (138). The increase in intracellular killing corresponded with an increase in granule/phagosome fusion and was mediated by a protein component in ALF, as the observed increase in killing was lost when ALF was heat-inactivated (138). Incubation with ALF also reduced extracellular degranulation in response to *M. tuberculosis* (138). Of note, neutrophils infected with ALF-treated *M. tuberculosis* did not activate macrophages and infection with ALF-treated *M. tuberculosis* had no significant effect on neutrophil apoptosis or necrosis. This study shows that exposure of *M. tuberculosis* to ALF alters the interaction of the bacteria with neutrophils in a way that facilitates neutrophils to kill the bacteria intracellularly without release of damaging neutrophil proteins.

Taken together, the evidence so far indicates that neutrophil oxidants are dispensable for killing of some mycobacteria. By dispensable we mean that oxidants are likely to contribute when they are being produced, but in their absence the non-oxidative mechanisms are able to compensate. More studies are required, ideally using neutrophils from CGD patients, to examine neutrophil killing of a wider range of mycobacteria. To our knowledge the question of whether neutrophils kill *M. leprae* is unanswered, and more studies are also needed to examine neutrophil killing of the more significant NTM, particularly MAC, *M.* *kansaii* and *M. abscessus*.

## Neutrophil Extracellular Trap-Mediated Killing of Mycobacteria

NETs contain proteins with antimicrobial activities, including MPO, calprotectin and elastase (12, 13, 169). Histones on NETs have also been shown to mediate microbial killing as has NET-DNA (9, 14, 170). The prolonged presence of cytotoxic NET constituents as a result of excessive NET release or impaired clearance can also be detrimental to the host.

*In vivo*, NET markers have been measured in the plasma of patients with active tuberculosis (171, 172) and observed to decrease with antibiotic therapy (171). NETs have also been observed in BALF samples from mice 3 – 4 weeks after aerosol challenge with *M. tuberculosis* (119) and in skin samples from guinea pigs within several hours following intradermal *M. tuberculosis* inoculation (173), indicating that NETs may participate in both early and late stages of infection. Whether their presence is important in control of *M. tuberculosis* infection or if they contribute towards pathogenesis remains to be determined. Recently, NETs have been observed in lung lesions resected from patients with persistent pulmonary TB and from TB-susceptible mice lending support to their role in TB pathogenesis (174). Increased human DNA-histone complexes associated with NETs have been measured in ENL sera compared with lepromatous and borderline lepromatous leprosy patients (175). NET components (DNA/histone/MPO) were observed in ENL skin lesions, and DNA-histone complexes in patient sera were significantly higher than those with lepromatous and borderline lepromatous leprosy and healthy controls (176). This data is suggestive of NETs contributing towards more severe leprosy disease. A recent review discusses the potential for NETs as a putative prognostic tool in ENL to direct medical treatment (177).

Several mycobacteria, including *M. tuberculosis*, *M. bovis* BCG, *M. abscessus, M. avium* subsp. *paratuberculosis* and *M. leprae*, have been shown to induce NETs *in vitro* (121, 129, 146, 148, 176, 178–181). How the interaction of mycobacteria with neutrophils leads to the formation of NETs appears to differ depending on the bacterial species. The early secreted antigen-6 (ESAT-6) of *M. tuberculosis* has been shown to induce neutrophils to release NETs (148, 181, 182), as has secreted sphingomyelinase Rv088 (182, 183). Rv088 has both sphingomyelinase (184) and nuclease (185) activity but it is the sphingomyelinase activity that is required for neutrophils to form NETs (183). Phagocytosis is also a prerequisite for the induction of NETs in response to *M. tuberculosis* and *M. abscessus*, as NET formation did not occur when phagocytosis was inhibited with cytochalasin D (146, 181). In contrast, Branzk et al. found with *M. bovis* BCG that NETs only occurred in response to small non-phagocytosed aggregates (179). Single bacteria were phagocytosed and neutrophils containing these bacteria did not go on to form NETs, at least not over the four hours of this study (179).

Neutrophil oxidants are required for NET formation in response to various stimuli (186–188), and *M. bovis* BCG NET induction was shown to be dependent on neutrophil oxidants (189). In addition, NETs were not formed under hypoxia in response to *M. tuberculosis* (190). Both rough and smooth morphotypes of *M. abscessus* were found to induce NETs, though the mechanism differed between morphotypes (146). Early NET induction (up to one hour) was independent of neutrophil oxidant production, while NETs formed after four hours co-incubation with bacteria did depend on oxidants, similar to that which has been reported for *S. aureus* (191).

Despite increasing evidence that mycobacteria induce neutrophils to form NETs, there is relatively little information on whether NETs contribute to killing of mycobacteria or control of infection. Evidence suggests that *M. abscessus* are killed by NETs whereas *M. tuberculosis* are not (146, 148, 173). In a study by Ramos-Kichik et al., *M. tuberculosis* was not killed by NETs formed by PMA (148). NET constituents as well as their post-translation modifications can vary depending on the stimulus (11, 181) potentially altering their bactericidal activity. Therefore, it is important to examine NETs induced either by the bacterium studied or host/bacterial factors that may be present at the infection site. In a guinea pig model of extra-pulmonary tuberculosis, NETs induced by *M. tuberculosis in vivo* were not bactericidal (173). This provides good evidence that NETs do not contribute towards killing of *M. tuberculosis*, but further studies are required to corroborate this in human disease.

NET-mediated killing of *M. abscessus*, as with NET induction, appears to differ with the morphotype studied. Neutrophil killing of the smooth morphotype was found to occur predominately *via* NETs, as removal with DNase resulted in only slight killing (146). In contrast, approximately half the rough morphotype were still killed when NETs were degraded, indicating other neutrophil killing mechanisms are more important for this morphotype. Notably in this study, neutrophil killing was only measured over the first hour of co-incubation when NET release was relatively low making it difficult to gain a full appreciation of the effect of NETs on *M. abscessus* viability. As the mechanism of NET formation differed between early and later formed NETs, it is conceivable that later-formed NETs may contain different constituents and therefore different antimicrobial activity.

There is evidence that NETs may increase the anti-mycobacterial capacity of macrophages. In one study, NETs produced in response to *M. tuberculosis* were shown to activate macrophages, leading to production of pro-inflammatory cytokines (181). In another study, *M. bovis* BCG was shown to induce NETs that contain the antimicrobial cathelicidin LL37, and macrophages were observed to ingest NET fragments containing this cathelicidin (189). By creating DNA : LL37 complexes to mimic NET fragments, Stephan et al. monitored the intracellular localization of these complexes in macrophages that had already phagocytosed BCG. Following uptake, the DNA was degraded in lysosomes releasing LL37 in close proximity to the internalized bacteria and inhibiting bacterial growth (189). Interestingly, significantly greater growth inhibition was observed when infected macrophages were incubated with DNA : LL37 complexes rather than LL37 alone. The authors speculated that the binding and internalization of DNA may increase killing by activating intrinsic antimicrobial pathways within the macrophage.

NETs may play a beneficial role in mycobacterial infection simply by capturing bacteria and preventing dissemination, and by facilitating activation of macrophages and other immune cells. Indeed, NETs have been shown to prime T cells (192). NETs are observed in the leprosy reaction ENL. However, evidence of free bacteria in ENL patient sera suggests that NETs are not capable of containing *M. leprae*. Moreover, NETs may also be detrimental. They may bind other immune cells inhibiting their function, and may also directly damage host tissue. NETs are linked to lung injury in respiratory conditions such as cystic fibrosis, chronic obstructive pulmonary disease, and pneumonia-associated acute respiratory distress syndrome (193–195). They have also been linked to lung injury in mice infected with *M. smegmatis* expressing the sphingomyelinase/nuclease Rv088 from *M. tuberculosis* (183). Lung injury was largely mediated by MPO (183), which is present and active on NETs (13). If NETs are proven ineffective at limiting infection, then there may be an advantage in blocking production or removing them to help protect damage to lung tissue.

## Mycobacterial Resistance to Neutrophil Oxidants

Killing of mycobacteria by neutrophils appears to be much slower than other pathogens, suggesting that these bacteria possess an innate resistance to the fast-acting oxidative killing mechanisms in the phagosome. In support of this, we have shown that *M. smegmatis* can cope with relatively high doses of the most bactericidal oxidant produced in the neutrophil phagosome, HOCl (LD_50_ of 90 nmol/10^8^ CFU) (127). HOCl reacts rapidly with a wide range of biomolecules, and the relative resistance may be related to the larger size of these microbes i.e. more HOCl is required to damage enough critical targets in the bacterium. A rod-shape *M. smegmatis* 10 µm in length and 0.8 µm in diameter has a volume of 5x10^-12^ mL. In contrast, *S. aureus*, *P. aeruginosa* and *E. coli* (rod- or sphere-shaped with 1-2 µm in length and 0.5–1 µm diameter) have approximately 10% that volume. The susceptibility of these bacteria to HOCl ranges from 2.5-3 nmol/10^8^ CFU for *P. aeruginosa* and *E. coli* (196, 197), to approximately 10 nmol/10^8^ CFU for *S. aureus* (151, 197, 198).

The presence of protective compounds will also contribute to HOCl resistance. Mycothiol (MSH), the main low molecular weight thiol (LMWT) in mycobacteria, has been proposed to play a role in defending against neutrophil oxidants. Mycothiol carries out many of the cellular functions performed by glutathione in eukaryotic cells and gram-negative bacteria, including the detoxification of electrophilic compounds and maintaining redox homeostasis (199). Interestingly, MSH levels in *M. tuberculosis* and *M. smegmatis* are much higher than those of the main LMWT in other bacteria such as bacillithiol (BSH) in *S. aureus* or glutathione in *Pseudomonas aeruginosa* (9–19, 0.7 and 1.1 µmol/g residual dry weight, respectively) (200). Since HOCl reacts very rapidly with thiol moieties (201), the higher LMWT content in mycobacteria might protect them from killing by HOCl. Consistent with a role for mycothiol in HOCl resistance, mycobacteria lacking the ability to synthesize this LMWT are significantly more sensitive to reagent HOCl and its secondary oxidants, chloramines (127, 202, 203). However, the amount of HOCl required to kill *M. smegmatis* (200-300 nmol/10^8^ bacteria) greatly exceeds that of mycothiol (5 nmol/10^8^ bacteria) (127), suggesting that mycothiol exerts its protective role not by scavenging the oxidant directly, but by forming mixed disulfides with critical cysteine residues in proteins in a process termed S-mycothiolation. Because S-mycothiolated proteins can be reduced by mycoredoxin-1 (203), this modification protects cysteine residues from irreversible oxidation. S-mycothiolation occurs in *M. smegmatis* exposed to hypochlorous acid (202). We recently observed that neutrophils killed mycothiol-deficient *M. smegmatis* at the same rate as wild type bacteria, indicating that mycothiol itself is not responsible for the ability of *M. smegmatis* to cope with HOCl or other oxidants produced in the phagosome (127).

Mycobacteria also contain other LMWTs such as gamma-glutamylcysteine, coenzyme A, cysteine and ergothioneine, albeit at much lower levels than mycothiol (127, 204). Interestingly, unlike mycothiol, ergothioneine is known to be actively exported suggesting an extracellular function for this LMWT (205), which may provide greater protection against HOCl. Also, ergothioneine, which is upregulated in mycothiol-deficient mutants, can compensate for the loss of mycothiol in protecting against organic hydroperoxides and is essential for survival of *M. tuberculosis* in macrophages and mice (205). The contribution of ergothioneine and other LMWTs to surviving neutrophil phagocytosis remains unexplored and future investigations are needed to establish their role in mycobacterial resistance to neutrophil oxidants. Apart from thiol groups, HOCl also reacts rapidly with methionine residues on proteins (201) resulting in the formation of methionine sulfoxide. Methionine sulfoxides are reduced by methionine sulfoxide reductases, the lack of which in *M. tuberculosis* made the bacteria more susceptible to HOCl (206). Whether or not methionine sulfoxide reductase activity protects mycobacteria from oxidative killing by neutrophils remains to be investigated.

Bacterial superoxide dismutase (SOD), which catalyzes the conversion of superoxide to hydrogen peroxide, is another candidate for conferring resistance of mycobacteria to neutrophil killing. SOD is exported in large amounts by *M. tuberculosis* and production increases further under hydrogen peroxide stress (207, 208). A role for SOD in the virulence of *M. tuberculosis* was established in a mouse infection model (207). Superoxide is the most-upstream of the oxidants produced by the neutrophil, and while SOD will facilitate conversion to hydrogen peroxide, the superoxide itself can modulate MPO activity (209). The addition of SOD to the surface of *S. aureus* slowed the rate at which they were killed by neutrophils (210). Non-pathogenic *M. smegmatis* express almost 100-fold less SOD than *M. tuberculosis* and export a smaller fraction (208). However, we could not slow the killing of *M. smegmatis* by genetically-modifying them to express large amounts of *M. tuberculosis* SOD, albeit as noted above, wild-type *M. smegmatis* are already killed very slowly (127).

## Conclusions and Future Perspectives

While neutrophils attempt to control mycobacterial infection, the bulk of the evidence indicates that the effectiveness of their phagosomal and extracellular killing mechanisms is thwarted by the microbes. Rather than destroy mycobacteria, the neutrophils appear to provide a safe haven and transport them into macrophages, while retaining the potential to damage host tissue. With regards to the latter, it will be valuable to determine if NETs play a significant role in the control of infection by preventing the spread or directly killing mycobacteria, or if they simply cause tissue damage and contribute towards a pro-inflammatory environment.

It is important to note the limitations of current experimental models (211). *In vitro* studies of neutrophil and mycobacteria interactions occur using neutrophils isolated from circulating blood; they have not been exposed to the plethora of signals and cell interactions that occur during migration and upon arrival at a site of infection. Human tuberculosis granulomas are highly hypoxic (212), which will suppress the oxidative burst, yet little is known about how hypoxia affects the response of neutrophils to mycobacteria (213). There is considerable heterogeneity in neutrophil populations, and certain subpopulations may be more effective at ingesting and destroying mycobacteria. Also, a significant amount of information has been derived from animal models, but neutrophils function differently between species, particularly mouse and human (214).

The current challenge is to apply our knowledge of neutrophil-mycobacteria interactions to improving treatments. The appearance of multi-drug resistance strains of *M. tuberculosis* is of major concern. Treatment for drug-susceptible *M. tuberculosis* involves six months of antibiotic therapy provided by four front line drugs: isoniazid, rifampicin, ethambutol and pyrazinamide. Treatment of infections with multi-drug resistant (MDR) strains takes longer, costs considerably more and uses second generation drugs with greater side effects and more complex drug delivery (2). Extensively drug-resistant strains have also arisen that are resistant to at least one second-line drug used to treat MDR (fluoroquinolone) and one second-line injectable drug (2). Infections with NTM can also be difficult to treat (215, 216).

In terms of bacterial resistance, the mycobacteria tested so far appear to be phagocytosed and killed more slowly than other pathogenic bacteria, with resistance to neutrophil oxidants likely to be an important factor. While a number of mechanisms have been shown to underwrite the resistance of mycobacteria to individual oxidants, none of these have yet been conclusively demonstrated to play a role in resistance to neutrophils. Better understanding of how these microbes survive oxidant exposure in the phagosome may provide therapeutic targets for sensitizing pathogenic mycobacteria to killing by the immune system. Pharmacological interventions might also be useful in limiting any adverse effects of NETs during mycobacterial infection.

## Author Contributions

HP compiled the first draft of the review, using additional content provided by LF, CK, and ND. MH edited the article and generated the final version. All authors contributed to the article and approved the submitted version.

## Funding

This work was supported by the Canterbury Medical Research Foundation, the Health Research Council of New Zealand (15/479), and the Travis Trust of New Zealand.

## Conflict of Interest

The authors declare that the research was conducted in the absence of any commercial or financial relationships that could be construed as a potential conflict of interest.

## Publisher’s Note

All claims expressed in this article are solely those of the authors and do not necessarily represent those of their affiliated organizations, or those of the publisher, the editors and the reviewers. Any product that may be evaluated in this article, or claim that may be made by its manufacturer, is not guaranteed or endorsed by the publisher.
